# Microdiversity of an Abundant Terrestrial Bacterium Encompasses Extensive Variation in Ecologically Relevant Traits

**DOI:** 10.1128/mBio.01809-17

**Published:** 2017-11-14

**Authors:** Alexander B. Chase, Ulas Karaoz, Eoin L. Brodie, Zulema Gomez-Lunar, Adam C. Martiny, Jennifer B. H. Martiny

**Affiliations:** a Department of Ecology and Evolutionary Biology, University of California, Irvine, California, USA; b Earth and Environmental Sciences, Lawrence Berkeley National Laboratory, Berkeley, California, USA; c Department of Earth System Science, University of California, Irvine, California, USA; University of Oklahoma

**Keywords:** *Actinobacteria*, *Curtobacterium*, *Microbacteriaceae*, drought, ecotypes, glycoside hydrolases, nitrogen addition

## Abstract

Much genetic diversity within a bacterial community is likely obscured by microdiversity within operational taxonomic units (OTUs) defined by 16S rRNA gene sequences. However, it is unclear how variation within this microdiversity influences ecologically relevant traits. Here, we employ a multifaceted approach to investigate microdiversity within the dominant leaf litter bacterium, *Curtobacterium*, which comprises 7.8% of the bacterial community at a grassland site undergoing global change manipulations. We use cultured bacterial isolates to interpret metagenomic data, collected *in situ* over 2 years, together with lab-based physiological assays to determine the extent of trait variation within this abundant OTU. The response of *Curtobacterium* to seasonal variability and the global change manipulations, specifically an increase in relative abundance under decreased water availability, appeared to be conserved across six *Curtobacterium* lineages identified at this site. Genomic and physiological analyses in the lab revealed that degradation of abundant polymeric carbohydrates within leaf litter, cellulose and xylan, is nearly universal across the genus, which may contribute to its high abundance in grassland leaf litter. However, the degree of carbohydrate utilization and temperature preference for this degradation varied greatly among clades. Overall, we find that traits within *Curtobacterium* are conserved at different phylogenetic depths. We speculate that similar to bacteria in marine systems, diverse microbes within this taxon may be structured in distinct ecotypes that are key to understanding *Curtobacterium* abundance and distribution in the environment.

## IMPORTANCE

Despite the plummeting costs of sequencing, characterizing the fine-scale genetic diversity of a microbial community—and interpreting its functional importance—remains a challenge. Indeed, most studies, particularly studies of soil, assess community composition at a broad genetic level by classifying diversity into taxa (OTUs) defined by 16S rRNA sequence similarity. However, these classifications potentially obscure variation in traits that result in fine-scale ecological differentiation among closely related strains. Here, we investigated “microdiversity” in a highly diverse and poorly characterized soil system (leaf litter in a southern Californian grassland). We focused on the most abundant bacterium, *Curtobacterium*, which by standard methods is grouped into only one OTU. We find that the degree of carbohydrate usage and temperature preference vary within the OTU, whereas its responses to changes in precipitation are relatively uniform. These results suggest that microdiversity may be key to understanding how soil bacterial diversity is linked to ecosystem functioning.

## INTRODUCTION

Currently, most studies assessing the responses of bacterial communities to environmental change rely on broad taxonomic designations, for instance, by using operational taxonomic units (OTUs) based on the nucleotide sequence similarity of the 16S rRNA gene (1). While this classification of bacterial diversity can capture broad taxonomic shifts, it provides limited genetic resolution at this loosely defined species level (2–4) by obscuring important genetic diversity within the OTU (5–7)—so-called microdiversity (8, 9). Given that most studies investigate microbial composition using 16S rRNA gene-defined OTUs (specifically, at the 97% level), a large gap in our understanding is the extent of microdiversity in natural communities and its relationship to variation in bacterial traits.

Growing evidence indicates that the genetic variation encompassed by bacterial microdiversity corresponds to variation in a wide range of functional traits (10). At fine genetic scales (11, 12), microbes with distinct physiological traits may partition niche space within the environment (13, 14). For example, extensive work in marine systems has demonstrated that microdiversity within a 16S rRNA gene-defined taxon encompasses distinct ecotypes, or lineages that respond differently to variation in the environment over space and time (14–17). However, our ability to characterize ecotypes at fine taxonomic levels is still largely dependent on cultured organisms because of the need to link genomic variation to phenotypic variation (18). Also, while metagenomic sequencing has advanced the identification of uncultivated organisms (19), the functional role of microdiversity has rarely been considered in soils, as we lack cultured representatives of microbes that are abundant in soil.

Diverse bacterial and fungal communities on leaf litter, the top layer of soil, play a key role in the carbon cycle. Litter decomposition mediates the loss of carbon through respiration to the atmosphere or its storage as organic matter in soil (20). The Loma Ridge global climate experiment (LRGCE) in southern California was established to test how future changes in precipitation and nitrogen availability may alter semiarid grassland and coastal sage scrub ecosystems. In grasslands at the LRGCE, the litter microbial community is dominated by bacteria (21), suggesting that bacteria perform the bulk of grassland litter decomposition. Over a 2-year period, the leaf litter community responded weakly but significantly to treatment manipulations (22, 23). At the 97% OTU level, a *Curtobacterium* OTU (phylum *Actinobacteria*, family *Microbacteriaceae*) was the most abundant taxon within the bacterial community (23). An analysis of *Curtobacterium* sequences from around the globe revealed the genus to be a cosmopolitan terrestrial taxon, with isolates derived primarily from plant and soil habitats (24). Further, genomic sequencing of *Curtobacterium* strains isolated from leaf litter indicated that the genus has a high genomic potential to decompose polymeric carbohydrates such as starch, cellulose, and xylan that are abundant in leaf litter (24).

Our previous work demonstrated that isolates belonging to *Curtobacterium* harbored extensive genomic diversity despite being clustered within a single OTU as defined by 16S rRNA (24). Here, we ask the following questions. (i) What is the extent of *Curtobacterium* microdiversity in a natural leaf litter bacterial community? (ii) Does this microdiversity encompass genetic and physiological variation in ecologically relevant traits? To address these questions, we used a combination of environmental field data and physiological lab assays to assess the distribution of traits within *Curtobacterium* and their phylogenetic conservatism. First, we examined the response of *Curtobacterium* microdiversity to manipulations of precipitation and nitrogen availability by using cultured isolates to inform metagenomic data. Moisture limitation, in particular, is likely a major stressor on litter bacteria in Southern California, which experiences long dry seasons with little rainfall. Second, we assayed both the genomic potential and metabolic capacity of isolates to depolymerize cellulose and xylan. As leaf litter is primarily composed of these polysaccharides, access to this primary carbon supply in this environment may be a highly advantageous trait.

## RESULTS

### *Curtobacterium* abundance and microdiversity.

We characterized *Curtobacterium* abundance and its microdiversity at the Loma Ridge global climate experiment (LRGCE) using 48 metagenomic sequence libraries from litter samples collected over a 2-year period. To estimate its relative abundance within the bacterial community, we created a custom pipeline using a curated reference database of 3,019 genomes representing 1,464 bacterial genera, including 16 *Curtobacterium* genomes (see Fig. S1 in the supplemental material). We calculated taxonomic abundance by using a phylogenetic classification of the metagenomic reads against the reference phylogeny, which we constructed using single-copy marker genes (25). Using our pipeline, we identified *Actinobacteria* and *Microbacteriaceae* as the most abundant phylum (46.3%) and family (28.2%), respectively. We detected similar relative abundances using MG-RAST annotations of the marker genes (Text S1; Table S1), but this approach did not detect any *Curtobacterium*. Therefore, we used the new pipeline to investigate finer taxonomic levels. This analysis revealed that *Curtobacterium* was the most abundant genus observed over the 2-year period within the leaf litter community, comprising an average of 7.8% of the bacterial community. However, even with our pipeline, we were unable to characterize 31.2% of the marker genes at or below the genus level.

10.1128/mBio.01809-17.1 TEXT S1  Comparison of bacterial community analyses. Download TEXT S1, DOCX file, 0.1 MB. Copyright © 2017 Chase et al. 2017 Chase et al. This content is distributed under the terms of the Creative Commons Attribution 4.0 International license.

10.1128/mBio.01809-17.2 FIG S1  Reference database constructed from >3,000 genomes representing the entire *Bacteria* domain (two genomes per genus). Multilocus phylogenetic analysis is unrooted and based on a concatenated alignment of 29 single-copy marker genes. Download FIG S1, TIF file, 3.1 MB. Copyright © 2017 Chase et al. 2017 Chase et al. This content is distributed under the terms of the Creative Commons Attribution 4.0 International license.

10.1128/mBio.01809-17.7 TABLE S1  Average taxonomic relative abundance of the bacterial communities across the metagenomic samples from 2010 to 2012 at the LRGCE. The values are average percent taxonomic relative abundance ± 1 standard deviation (SD). Download TABLE S1, DOCX file, 0.1 MB. Copyright © 2017 Chase et al. 2017 Chase et al. This content is distributed under the terms of the Creative Commons Attribution 4.0 International license.

**FIG 1:**
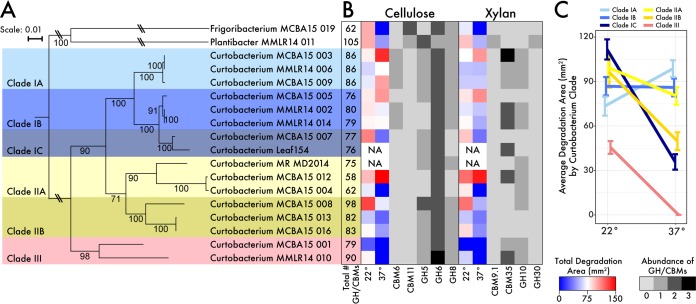
Phylogeny and traits of *Curtobacterium* strains. (A) Multilocus phylogenetic analysis using a concatenated alignment of 29 single-copy marker genes. Bar, 0.01 amino acid substitutions per position. (B) Genomic and physiological metrics of carbohydrate utilization. The total number of GH/CBM families targeting all potential carbohydrate substrates is shown in the first column. The physiological ability to degrade cellulose and xylan is shown in blue or red, while the genomic potential (presence of GH/CBM families) to degrade either cellulose or xylan is represented in gray or black. Strains that were not assayed (NA) for carbon degradation are indicated. (C) Average degradation area (±1 standard deviation [SD] [error bar]) of the substrates by *Curtobacterium* clade at each temperature.

On the basis of the full-length 16S rRNA gene, the *Curtobacterium* genomes (14 of which were cultured isolates from leaf litter [24] and two other publically available genomes) clustered in the same OTU defined at the 97% sequence identity level. We therefore identified genomic clusters within the *Curtobacterium* OTU using a phylogenetic analysis of 29 single-copy marker genes and grouped the isolates into six well-supported clades (Fig. 1A). These clades were supported by nucleotide (average nucleotide identity [ANI]) and amino acid (average amino acid identity [AAI]) similarity (Table S2). Specifically, isolates shared >97% AAI within clades for the 29 marker genes. Across the whole genome, isolates within clades were more similar in ANI and AAI than isolates between clades, which had a minimum pairwise similarity of 83.2% ANI and 78.9% AAI across all *Curtobacterium* isolates.

10.1128/mBio.01809-17.8 TABLE S2  Genomic characterizations of all *Curtobacterium* isolates within individual clades. Download TABLE S2, DOCX file, 0.1 MB. Copyright © 2017 Chase et al. 2017 Chase et al. This content is distributed under the terms of the Creative Commons Attribution 4.0 International license.

**FIG 2:**
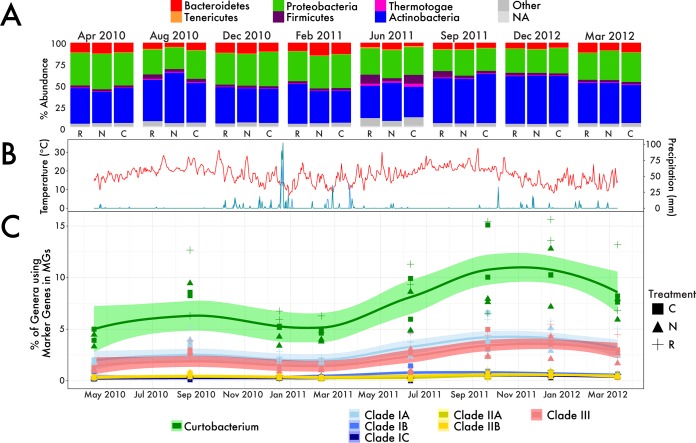
Bacterial community composition in the Loma Ridge field site over 2 years. (A) Relative abundances of the six most abundant phyla; replicates were averaged for each treatment and time point. The treatments were the addition of nitrogen (N), reduced precipitation (R), and control treatment (C). NA, not available. (B) Temperature and precipitation at Loma Ridge collected from May 2010 to March 2012. (C) Relative abundance of total *Curtobacterium* and each individual clade over time and by treatment. Smoothed averages (lines) were calculated from locally weighted smoothing (LOESS) with confidence intervals (colored areas). MGs, metagenomic sequences.

We then classified the metagenomic marker gene reads assigned to *Curtobacterium* in the taxonomic analysis onto the six identified clades. Only a tiny fraction (0.27% of the total bacterial community) of the bacteria identified as *Curtobacterium* failed to classify into one of the six clades, suggesting that our isolates encompassed most of the genomic diversity of *Curtobacterium* at the LRGCE. Across all samples, *Curtobacterium* was dominated by two clades (Table S1); clades IA and III averaged 3.0% and 2.4% of the marker gene sequences, respectively (Fig. 2C). Together, the remaining *Curtobacterium* clades (clades IB, IC, IIA, and IIB) composed >2% of the bacterial community, but separately, each of the four clades represented <0.6% of the bacterial community (Table S1).

### Response to the global change treatments.

Within the global change experiment, the composition of the microbial community varied seasonally by sampling date such that some bacterial phyla, including *Actinobacteria*, were strongly correlated with background precipitation (Fig. S2A) as previously reported (26). Indeed, at the phylum level (Fig. 2A), bacterial composition varied significantly over time (Bray-Curtis similarity; *P* < 0.002 by permutational multivariate analysis of variance [PERMANOVA]) and responded marginally to the global change treatments of reduced precipitation (drought) and added nitrogen (*P* = 0.061), with no significant interaction between the two factors. However, the global change treatments explained only 1.9% of the variation in phylum composition, whereas time (date of collection over a 2-year period) explained 65.1%. In particular, during a prolonged hot, dry season in the second year (Fig. 2B), the bacterial community became dominated by *Actinobacteria* (Fig. 2A).

10.1128/mBio.01809-17.3 FIG S2  Multidimensional scaling (MDS) plot depicting the difference in microbial community relative abundances at the phylum level (A) and clade level within *Curtobacterium* (B). Symbols represent sampling date with darker colors indicating winter/spring months and warmer colors indicating summer/fall months. Download FIG S2, TIF file, 2.6 MB. Copyright © 2017 Chase et al. 2017 Chase et al. This content is distributed under the terms of the Creative Commons Attribution 4.0 International license.

Much of the response of *Actinobacteria* to the global change treatments was due to *Curtobacterium*. The relative abundance of all *Curtobacterium* increased by 20.2% in the drought treatment and decreased by 17.2% in the nitrogen treatment relative to the control plots (*P* < 0.05 by PERMANOVA; Table S3). Similar to the phylum-level response, the time of sampling explained the greatest amount of variation in *Curtobacterium* abundance, accounting for 52.6% of the variation, while the treatment accounted for only 5.0%. *Curtobacterium* abundance was strongly associated with seasonal precipitation (Fig. S3A), increasing in relative abundance during the dry seasons and accounting for more than 10% of all leaf litter bacteria in the second, drier year of the study (Fig. 2B and C). *Curtobacterium* abundance, however, was not correlated with the mean temperature in the field 2 weeks prior to sampling (Fig. S3B).

10.1128/mBio.01809-17.4 FIG S3  Average relative abundance of *Curtobacterium* in the bacterial community at LRGCE as a function of the total precipitation (A) and the mean temperature (B) in the 2 weeks prior to sampling. Average relative abundance (± 1 SD) is shown. Symbols represent sampling date with darker colors indicating winter/spring months and warmer colors indicating summer/fall months. Download FIG S3, TIF file, 27 MB. Copyright © 2017 Chase et al. 2017 Chase et al. This content is distributed under the terms of the Creative Commons Attribution 4.0 International license.

10.1128/mBio.01809-17.9 TABLE S3  Relative abundance of *Curtobacterium* clades by treatment averaged across the metagenomic libraries from the LRGCE. Values are relative percent abundance ± 1 SD. Download TABLE S3, DOCX file, 0.1 MB. Copyright © 2017 Chase et al. 2017 Chase et al. This content is distributed under the terms of the Creative Commons Attribution 4.0 International license.

Next we tested whether microdiversity within *Curtobacterium* (and in particular, the six identified clades) varied in their responses to the global change treatments. All *Curtobacterium* clades responded similarly to drought, increasing in abundance relative to the control and, with the exception of clade IC, responded negatively to the increased nitrogen treatment (Table S3). Furthermore, *Curtobacterium* clade composition varied significantly over time (*P* < 0.001 by PERMANOVA; Fig. S2B), with clades IA and III increasing in relative abundance during the drier, second year of the study (Fig. 2C).

### Carbohydrate degradation traits within *Curtobacterium*. (i) Genomic characterization.

To analyze the genomic potential for carbohydrate degradation, we characterized the glycoside hydrolase (GH) and carbohydrate binding module (CBM) protein families within and among *Curtobacterium* clades. The abundance of total GH and CBM (GH/CBM) genes varied among all genomes, ranging from 58 to 98 GH/CBM copies. The total distribution of GH/CBM genes varied significantly with phylogenetic distance such that more closely related genomes carried more similar copy numbers (RELATE test; ρ = 0.45 and *P* < 0.05; Fig. S4). Clades IA, IIB, and III encoded the highest abundance of GH/CBM genes (an average of 86, 87.7, and 84.5 genes), which differed significantly (*F*^5,10^ = 5.3027 by analysis of variance [ANOVA]; *P* < 0.05) from clade IIA (65 genes), whereas clades IB and IC encoded intermediate numbers of these genes (76.5 and 78.3, respectively; Fig. 1B).

10.1128/mBio.01809-17.5 FIG S4  Multidimensional scaling (MDS) plot depicting total GH/CBM gene composition for *Curtobacterium* isolates. Each symbol represents a genomic isolate and is colored according to the assigned clade. Download FIG S4, TIF file, 16.3 MB. Copyright © 2017 Chase et al. 2017 Chase et al. This content is distributed under the terms of the Creative Commons Attribution 4.0 International license.

Next, we considered the GH/CBM gene diversity that is thought to be responsible for degradation of the most abundant carbohydrates in the leaf litter at the LRGCE, cellulose and hemicellulose (specifically, xylan) (22). Overall, the numbers of both cellulose- and xylan-related GH/CBMs were significantly correlated with phylogenetic distance (RELATE test; ρ = 0.57 and *P* < 0.01 and ρ = 0.26 and *P* < 0.05, respectively). All *Curtobacterium* genomes contained at least one copy of a GH or CBM protein family that targeted either cellulose or xylan. However, some strains (e.g., MCBA15013 and MCBA15016, both from clade IIB) had an elevated abundance of GH/CBM genes targeting cellulose, with an apparent absence of genes targeting xylan. Clades IA and IB were the only clades to contain both GH and CBM genes targeting each substrate (Fig. 1B).

### (ii) Phenotypic characterization.

The presence of GH/CBM genes within a genome suggests only the potential for substrate utilization. Therefore, we conducted substrate assays in the laboratory to confirm the ability of each isolate to degrade cellulose and xylan at 22°C. We performed these assays at this temperature, as the optimum growth for the genus is thought to range from 20 to 26°C (27, 28). All but one of the strains (MCBA15001) degraded both cellulose and xylan over a 4-day period, including the four isolates that did not carry known xylan-targeting genes (Fig. 1B). Indeed, the size of an isolate’s zone of depolymerization was not correlated with the abundance of genes targeting either cellulose (*F*^1,14^ = 1.24 by phylogenetic independent contrast [PIC] analysis; *P* > 0.05) or xylan (*F*^1,14^ = 0.15 by PIC analysis; *P* > 0.05).

The degradation patterns of the *Curtobacterium* strains also depended greatly on the temperature of the assay. When isolates were assayed at 37°C, the expected maximum temperature for growth in *Curtobacterium* (28), four strains exhibited an increase in degradation capability, including two strains from clade IA, while three strains were unable to degrade either substrate at 37°C (Fig. 1B). The total area of the zone of depolymerization varied significantly by temperature (*F*^1,43^ = 4.67 by analysis of covariance [ANCOVA]; *P* < 0.05) and clade (*F*^5,43^ = 4.74; *P* < 0.01), with a significant interaction between them (*F*^5,43^ = 2.46; *P* < 0.05), whereas the assay substrate had no effect on the depolymerization area (*F*^1,43^ = 0.95; *P* > 0.05). When averaged across *Curtobacterium* clades, only clade IA saw an average increase in depolymerization area when strains were grown at 37°C compared to 22°C (Fig. 1C). Most clades maintained some level of degradation capability at the higher temperature except for clade III, which failed to depolymerize either cellulose or xylan at 37°C (Fig. 1C).

## DISCUSSION

In this study, we investigated the extent of genomic microdiversity of *Curtobacterium* in the field and the relationship between this diversity and the bacterium’s functional traits. To our knowledge, this study is the first to do so in a dominant soil bacterium. As in aquatic and host-associated ecosystems (6, 8, 14, 29–31), microdiversity within this abundant bacterium is extensive. Cooccurring strains within the same *Curtobacterium* OTU have an average nucleotide identity (ANI) of as low as 83%, far below the traditional species boundary (32). Our results support the growing understanding that traditional taxonomic assignments (i.e., OTUs) are insufficient to resolve ecologically distinct microorganisms (33, 34). Indeed, extensive *Curtobacterium* microdiversity persists in grassland leaf litter and encompasses variation in several ecologically relevant traits, including its ability to degrade abundant carbohydrates as well as temperature preferences for this degradation. Thus, binning 16S rRNA sequences obscures detection and interpretation of ecologically important trait variance.

Trait variability within soil bacterial OTUs has been described previously, suggesting that local adaptation and coexistence are probable among closely related strains (35–38). However, the combination of lab assays on cultured representative isolates in conjunction with metagenomic data allowed us to compare the physiological findings to their representation in the environment, as well as test the response to environmental change in the context of the whole community. Further, this combination enabled us to quantify and interpret metagenomic data of ecologically relevant microdiversity that would otherwise not be detected (Table S1) due to the absence of genomic representation in public databases. Indeed, particularly for terrestrial soil systems, the genomic reference databases often lack the resolution to detect fine-scale taxonomic groups, as defined by an ANI of >95% (39), or result in mischaracterization of taxonomic groups altogether (40).

The results of this study are also consistent with the idea that bacterial traits are often conserved at different phylogenetic depths (14, 41). Complex quantitative traits like an organism’s response to drought have been proposed to be more phylogenetically conserved (10, 41). Here, we observed that a response to dry conditions (both in the drought treatment and the dry seasons) appears to be generally consistent among *Curtobacterium* clades, suggesting that biological and physiological traits responsible for moisture response are ecologically cohesive (42) within this taxon. Thus, the response of *Curtobacterium* to future drought would likely be apparent at the OTU level, although certain clades may be more abundant than others. However, given that some of the clades were relatively rare within the community, further investigation is still needed to confirm this interpretation.

In contrast, traits that rely on one gene or a few genes such as carbon utilization are thought to be more shallowly conserved (43), as they may be more prone to horizontal gene transfer. Using physiological assays, we confirmed the genomic potential for *Curtobacterium* to degrade polymeric carbohydrates, which are likely central to their success within the leaf litter community. Although all *Curtobacterium* clades could depolymerize both xylan and cellulose, the degree of carbohydrate utilization varied among and within clades, suggesting that carbohydrate utilization is finely conserved. Such intricate differences in carbohydrate degradation traits among *Curtobacterium* may contribute to the persistence of this microdiversity within the leaf litter community. However, the genomic potential for carbohydrate utilization (number and composition of GH/CBMs) did not predict observed phenotypic variation in the lab, highlighting the difficulty in using gene annotations to predict ecological roles.

Carbohydrate degradation was also temperature dependent, regardless of the substrate. Further, this dependency varied among clades, revealing that *Curtobacterium* microdiversity also incorporates variation in temperature preference. Broadly, this result supports the idea that bacterial temperature preference can be relatively finely conserved (41), in agreement with rapid temperature adaptation observed in the lab (44). More specifically, it suggests differential physiological tradeoffs between temperature and carbohydrate utilization (45) among clades. Such variation in this tradeoff might explain the coexistence of these closely related clades, particularly for the two most abundant clades, clades IA and III. Despite similar environmental responses to drought and seasonal fluctuations, these clades exhibited opposite responses to temperature with respect to carbohydrate utilization (Fig. 1C). While temperature preference has previously been shown to drive shifts in ecotype abundance within marine systems (16), we did not observe a correlation between clade abundance and temperature at this one site. However, further investigation is needed across a wider temperature range to test whether temperature drives the geographic distribution of *Curtobacterium* clades.

In conclusion, the microdiversity within a single *Curtobacterium* OTU in this grassland leaf litter encompasses variation in traits involved in carbon degradation and temperature preference. Classic ecological theory would suggest that this trait variation allows microdiversity to occupy distinct ecological niches (46), although further work is needed to identify distinct *Curtobacterium* ecotypes in the environment. At the same time, *Curtobacterium* appears to be consistent in its response to changes in precipitation, suggesting that variability in moisture conditions are unlikely to explain the maintenance of this microdiversity. Thus, similar to marine bacteria (8, 9), our work highlights that the depth of trait conservatism (14) may help to understand the response of soil bacteria to changing environments.

## MATERIALS AND METHODS

### Field site.

The Loma Ridge global change experiment (LGRCE) (in Irvine, California, USA [33° 44′ N, 117° 42′ W]) (47) was established in 2007 with precipitation and nitrogen manipulations in areas of deciduous shrubland (coastal sage scrub) and annual grasses. For this study, we sampled only in the grassland plots, which are dominated by *Avena*, *Bromus*, and *Lolium* (22). We used a subset of the plots that included reduced precipitation treatment (50% reduction in annual precipitation), added nitrogen treatment (20 to 40 kg N/ha), and a control treatment, as previously described (22).

We collected leaf litter from these plots by sampling each season from May 2010 to March 2012 across three treatments: control, reduced precipitation (drought), and added nitrogen (8 time points × 3 treatments × 2 replicates). As described previously, metagenomic libraries were created from these samples by extracting DNA from ground litter, prepared using a TruSeq library kit (Illumina, San Diego, CA), and sequenced on an Illumina HiSeq2000 instrument. Samples were pooled from eight plots from each treatment to form the two replicate libraries at each time point (for more information, see reference 26). The sequence libraries are available on MG-RAST under the project identifiers (IDs) 4511045 to 4511050, 4511060 to 4511065, 4511111 to 4511116, 4511134 to 4511153, and 4511178 to 4511193. We excluded two libraries (Drought April 2010 and Nitrogen August 2010) due to low sequence count. Temperature and precipitation data were recorded at a nearby weather tower (22).

### Curated marker gene reference database.

We developed a reference genomic database to characterize phylogenetic marker genes from the metagenomic sequences of the microbial community. This approach is similar to PhyloSift (48), except we performed a more robust search to compensate for the lack of genomic references to characterize soil microbial communities. We downloaded 79,838 genomes from the PATRIC database (49) with RAST (50) annotations on 9 December 2016. We screened all genomes for annotations of 29 conserved, single-copy phylogenetic marker genes (25) and discarded failed genomes, most of which were draft genomes with >1,000 contigs. The remaining genomes were manually curated by assigned nomenclature to include two genomes per genus. We used complete genomes and genomes isolated from soil ecosystems when they were available. The 3,159 resulting genomes were combined with 14 *Curtobacterium* genome sequences isolated from two grassland leaf litter sites (24), including four strains isolated during the time of metagenomic sampling from the LRGCE.

We curated the downloaded genomes to ensure that all genomes were properly assigned to the correct taxon. Individual marker genes from each genome were aligned using ClustalO v1.2.0 (51) and used to construct a 15,963-bp concatenated alignment for phylogenetic analysis using FastTree 2 (52). The resulting reference phylogeny guided the construction of each individual marker gene tree to maintain relative node structure across trees. For each marker gene tree, we performed a maximum likelihood bootstrap analysis using RAxML v8.0.0 (53) under the PROTGAMMAWAGF model for 100 replicates. If a genome was named incorrectly or showed a problematic alignment for any of the individual marker gene trees (i.e., genome terminal branch length was >5), the entire genome was removed (a total of 154 genomes were removed), and all trees were regenerated.

The NCBI taxonomy database (54) was downloaded on 17 June 2016. The taxonomic information of the remaining 3,019 genomes was added locally to the NCBI database using the PATRIC genome IDs. The individual marker gene trees and taxonomic information were all used to generate reference packages for the program pplacer v1.1.alpha17 (55). Reference packages were subsequently used to characterize the microbial community (available at https://github.com/alex-b-chase/LRGCE).

### Metagenomic analyses.

To evaluate the taxonomic diversity of the bacterial community as well as finer-scale diversity within *Curtobacterium* at the LRGCE, we reanalyzed the metagenomic libraries previously described (26). Metagenomes were retrieved from the metagenomics analysis server (MG-RAST) (56) after sequences had been processed for quality control and coding regions were predicted by FragGeneScan (57). We performed an initial filter using BLASTP (58) against our custom database with an E value of 1 × 10^−5^. We applied a secondary filter using HMMER v3.1b2 (59) with an E value of 1 × 10^−10^ to achieve a higher specificity. We grouped the filtered reads for each library by each marker gene and aligned them using ClustalO v1.2.0 (51) to the corresponding marker gene reference package (see above). Aligned metagenomic reads were “placed” onto the reference phylogeny using pplacer v1.1.alpha17 (55), keeping at most 20 placements, and a posterior probability for final placement on the reference tree was calculated. Finally, we created single branch abundance matrices yielding an abundance distribution ranging from phyla to individual genomes. All abundances were normalized by the total number of marker genes present.

### Comparison of the curated pipeline to other methods.

To validate the taxonomic results generated by our custom pipeline (Text S1), we compared our taxonomic abundances using two alternative approaches: (i) the MG-RAST pipeline using a read-based analysis and (ii) a *de novo* coassembly of all metagenomic libraries using paired-end reads.

First, to generate the MG-RAST taxonomic profiles, we downloaded the Kyoto Encyclopedia of Genes and Genomes (KEGG) database annotations for each library from the MG-RAST API (56) and calculated relative abundances across all annotated reads. Next, we standardized the MG-RAST output by filtering the MD5 IDs corresponding to the 29 marker genes and regenerated standardized taxonomic abundance profiles. All gene sequences retrieved from MG-RAST were assigned to the closest hit genus in the MG-RAST database using an E value of 1 × 10^−5^.

Second, we conducted a genome-centric analysis by performing a *de novo* coassembly of all of the paired-end shotgun metagenomic libraries using MEGAHIT (60). We used an iterative k-step ranging from k = 27 to 111 and discarded all assembled contigs of <3,000 bp. Read coverage for each assembled contig was calculated using bbwrap.sh within the suite of tools available via BBMap v35.66 (61). Taxonomic assignments for all assembled contigs were generated using MegaBLAST against the NCBI nucleotide database (January 2015 version) with an E value of 1 × 10^−5^.

### Genomic comparisons of the isolates.

To validate that all *Curtobacterium* genomes, including two publically available *Curtobacterium* genomes, clustered within the same OTU, we used Barrnap (http://www.vicbioinformatics.com/software.barrnap.shtml) to predict rRNA genes and clustered the 16S rRNA gene using UCLUST (62). We then examined the relationship among all 16 *Curtobacterium* genomes using 29 single-copy phylogenetic marker genes (25). Each conserved gene was independently aligned using ClustalO v1.2.0 (51) and used to create a concatenated alignment for phylogenetic analyses. We constructed a maximum likelihood phylogenetic tree using RAxML v8.0.0 (53) under the PROTGAMMAWAGF model for 100 replicates. For convenience, we designated six monophyletic clades based on the results from the phylogenetic analyses. To confirm these designations, we calculated pairwise average amino acid identity (AAI) across the 29 marker genes across all genomes.

Next, we confirmed that our clade designations were in accordance with additional genomic characterizations. Specifically, we calculated pairwise whole-genome average nucleotide identity (ANI) and AAI (63) and computed the core genome for each clade by generating groups of orthologous proteins with MCL (64). Genes identified as orthologous groups within clades were subsequently used to calculate the AAI of all clade-specific core genes. All genomic analyses were performed using the suite of tools available in the Microbial Genomes Atlas (MiGA) (https://github.com/bio-miga/miga).

To analyze each genome for its potential to degrade carbohydrates, genomic open reading frames (ORFs) were generated by the RAST annotation pipeline (50) and searched using HMMER against the Pfam-A v30.0 database (65). We then used a subset of identified protein families, representing glycoside hydrolase (GH) and carbohydrate binding module (CBM) proteins to identify the genomic potential to degrade carbohydrates of each isolate (24, 66). GH and CBM (GH/CBM) gene composition profiles for each isolate were subsequently used to generate a Bray-Curtis similarity matrix to produce a nonmetric multidimensional scaling (MDS) ordination plot.

### Physiological analyses of the isolates.

In the laboratory, we characterized the 14 *Curtobacterium* isolates for their ability to utilize two polysaccharides, cellulose and xylan, at two temperatures. All isolates were grown from −80°C freezer stocks for 24 to 48 h in LB liquid medium at room temperature (22°C). Isolates were spun down at a relative centrifugal force (RCF) of 13,500 for 4 min, and the LB supernatant was discarded. Pelleted cultures were washed with 0.9% saline solution three times and resuspended in 10 ml of M63 minimal medium with 0.5% (wt/vol) dextrose and allowed to grow for 24 h. All cultures were then diluted to an optical density at 600 nm (OD_600_) of 0.1 to ensure equal cell density across isolates. We used 10 µl of the cultures grown (in triplicates) to inoculate solid M63 medium containing 0.5% (wt/vol) carboxymethyl cellulose (CMC) (catalog no. 150560; MP Biomedicals) or xylan (catalog no. X0502; Sigma) and placed at 22°C (optimum temperature for growth [27, 28]) and 37°C (maximum temperature for growth [28]). Depolymerization of each substrate was classified after 4 days by measuring the zones of transparent growth around the inoculum as previously described (67) with Gram’s iodine stain (68). We analyzed the zones of depolymerization around inoculated colonies on ImageJ (https://imagej.nih.gov/ij/) to calculate the total area of carbohydrate degradation. An *Escherichia coli* strain was included as a negative control for all physiological assays.

### Statistical analyses.

To test the effects of environmental treatment manipulations on the distribution of bacterial communities and *Curtobacterium* clade composition, we used a permutational multivariate analysis of variance (PERMANOVA) (69). The statistical model included plot treatment (control, drought, or N addition) and date of collection as fixed effects. We tested the effects of time and treatment by generating Bray-Curtis similarity matrices at the phylum and clade taxonomic levels. Subsequent PERMANOVA analyses used a type III partial sum of squares for 999 permutations of residuals under a reduced model. Similarity matrices were also used to generate nonmetric multidimensional scaling (MDS) ordination plots. All multivariate statistical analyses were conducted using PRIMER6 with the PERMANOVA+ function (Primer-E Ltd., Ivybridge, UK).

We analyzed the distribution of GH/CBM genes within and among *Curtobacterium* clades. To test for differences in the total abundance of GH/CBM proteins across clades, we used a one-way analysis of variance (ANOVA). For the ANOVA analysis, we used a Tukey’s honestly significant difference test to detect the difference in the total abundance of GH/CBM genes across clades. To test for correlations between the abundance of GH/CBM proteins, with respect to cellulose and xylan, and phylogenetic distance, we calculated a Spearman’s rank correlation coefficient using a RELATE test. Further, we performed a phylogenetic independent contrast (PIC) analysis to test whether the abundance of GH/CBM genes was related to an isolate’s phenotypic ability to degrade cellulose or xylan in the laboratory. Finally, to determine the factors driving degradation, we used a multiple regression model including the following variables: temperature, clade designation, and carbon substrate. Starting with a three-way analysis of covariance (ANCOVA), we implemented a backward selection process (70). If the model returned nonsignificant interactions, the interaction was removed, and the model was regenerated to decrease the chance of spurious relationships (71). All analyses were performed in the R software environment.

10.1128/mBio.01809-17.6 FIG S5  Coverage profiles of all coassembled contigs as a function of their GC content. Taxonomic assignments are colored by the top 10 most abundant bacterial genera (listed in descending order). Download FIG S5, TIF file, 4.3 MB. Copyright © 2017 Chase et al. 2017 Chase et al. This content is distributed under the terms of the Creative Commons Attribution 4.0 International license.
