# Supplementary material for: Microdiversity of an Abundant Terrestrial Bacterium Encompasses Extensive Variation in Ecologically Relevant Traits
Source: mBio. 2017 Nov 14;8(6):e01809-17. doi: 10.1128/mBio.01809-17 (PMC5686540; doi:10.1128/mBio.01809-17)
Supplement: TEXT S1 [file mbo006173588s1.docx]

**Supplementary Text**

Comparison of Bacterial Community Analyses

We characterized the leaf litter microbial community at LRGCE using 48 metagenomic sequence libraries from samples collected over a two-year period. A commonly-used metagenomic pipeline (MG-RAST) indicated that, across all samples, the bacterial community was dominated by three phyla: Bacteroidetes (8.3%), Proteobacteria (44.5%), and Actinobacteria (36.6%). Considering only 29 single-copy marker genes annotated by MG-RAST, these three phyla appeared even more dominant, composing 97.9% of the bacterial community. Across all samples, Actinobacteria was the most abundant phylum (55.4%) and Microbacteriaceae was the most abundant family (25.1%) of all bacterial sequences (Table S1). However, this pipeline provided insufficient taxonomic signal to characterize the bacterial community as typically performed. For example, *Curtobacterium* was absent from the MG-RAST annotations, despite being previously identified as the most abundant genus in the leaf litter community (Table S1).

Additionally, we performed a genome-centric analysis by co-assembling all 48 of the metagenomic libraries. The assembly generated a total of 87,430 contigs >3000 bp (N50=5380 bp), representing 479 distinct bacterial genera (Fig. S5). Using MegaBLAST to characterize the contigs, the most abundant taxon belonged to *Sphingomonas* (family: Sphingomonadaceae), which constituted 6.8% of all contigs >3000 bp. Despite the low representation of *Curtobacterium* in the NCBI database (8 publically available genomes at the time of analysis), *Curtobacterium* (3.3%) was the third most abundant taxon behind *Sphingomonas* and *Clavibacter* (3.5%; family: Microbacteriaceae). Since this approach only used a best-hit taxonomic assignment with a moderately-conservative e-value (1x10^-5^), we suspected that *Curtobacterium* was still under-represented, particularly with regards to other Microbacteriaceae taxa. Indeed, 57.24% of assembled contigs could not be annotated to any assigned bacterial genera in the NCBI database. While conducting this genome-centric analysis, we confirmed *Curtobacterium* to be an abundant bacterium. However, due to the complexity of our bacterial community (Fig. S5), we were unable to segregate contigs among closely related strains to accurately distinguish *Curtobacterium* microdiversity.
