# Supplementary figures and images for: Microdiversity of an Abundant Terrestrial Bacterium Encompasses Extensive Variation in Ecologically Relevant Traits
Source: mBio. 2017 Nov 14;8(6):e01809-17. doi: 10.1128/mBio.01809-17 (PMC5686540; doi:10.1128/mBio.01809-17)

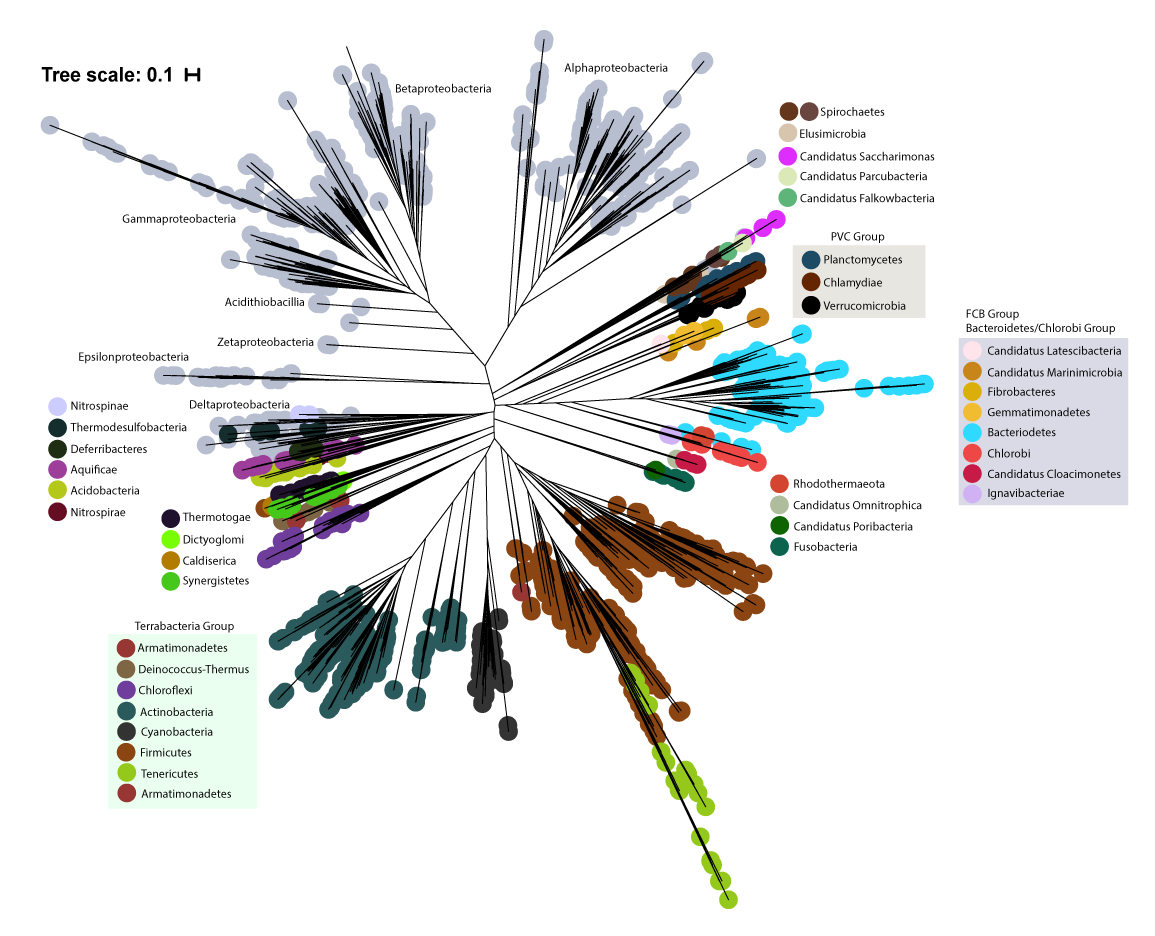

Supplement: FIG S1 [file mbo006173588sf1.tif]

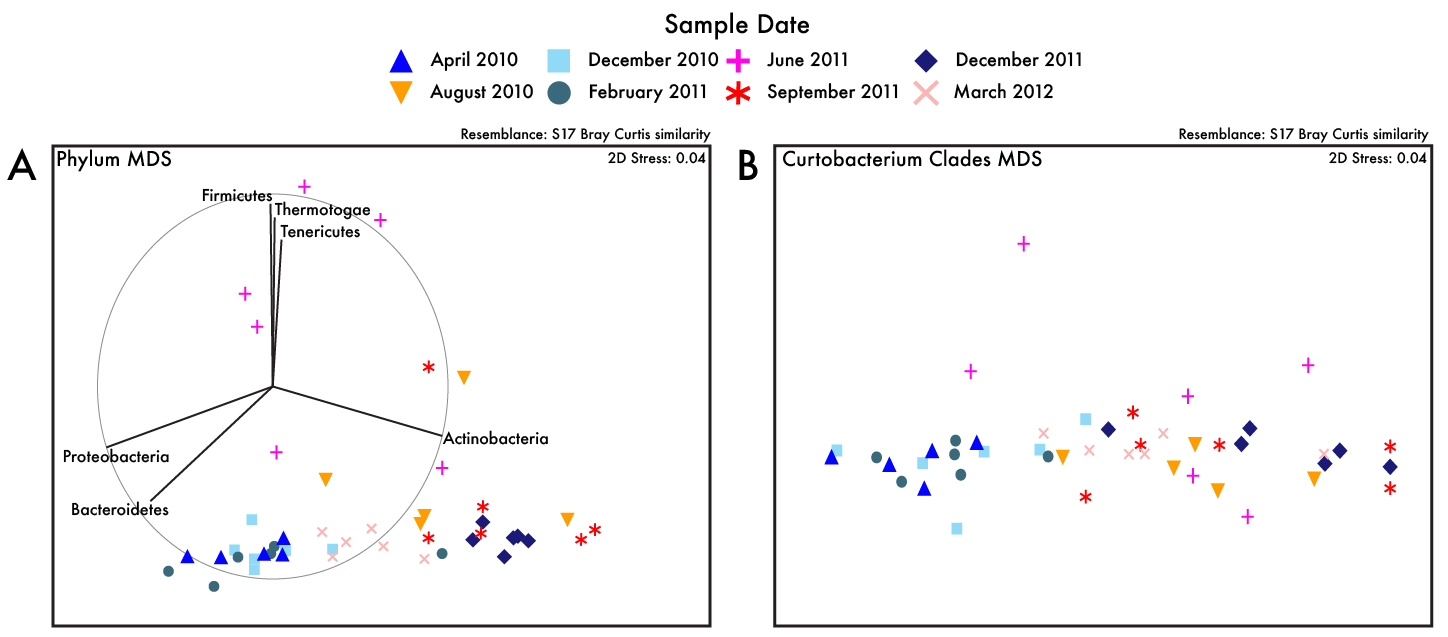

Supplement: FIG S2 [file mbo006173588sf2.tif]

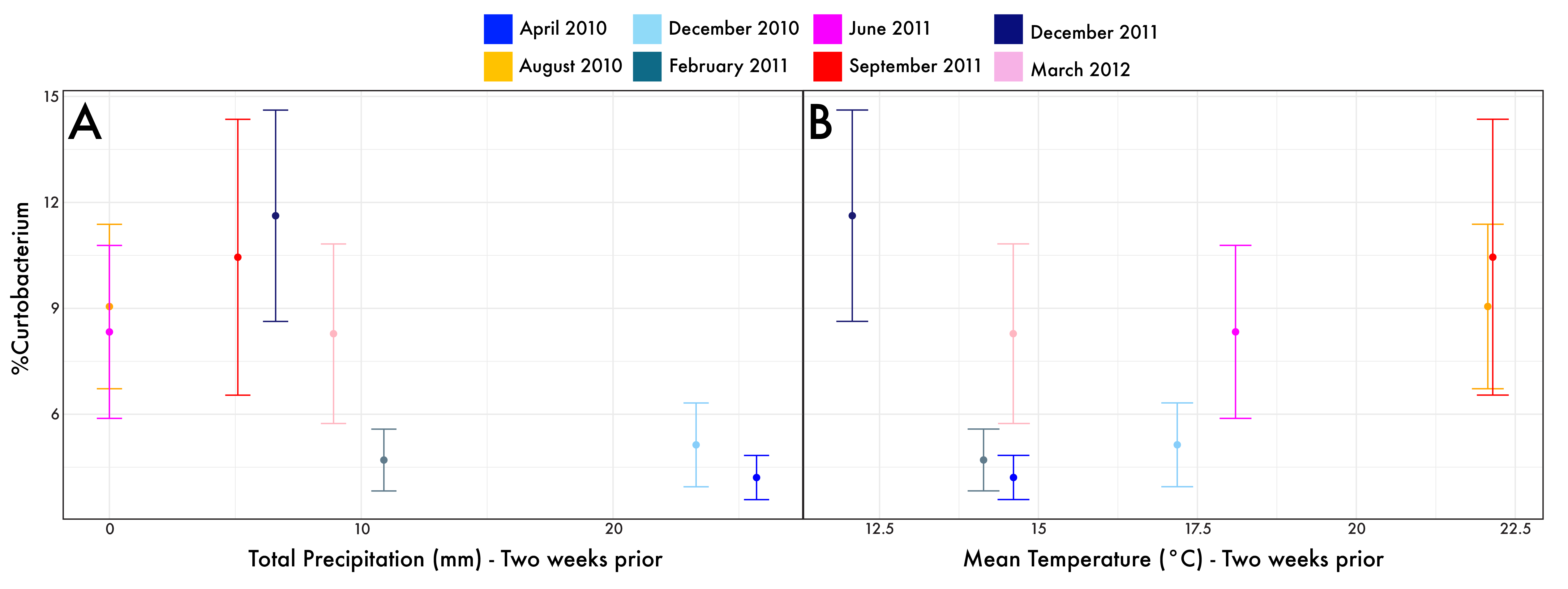

Supplement: FIG S3 [file mbo006173588sf3.tif]

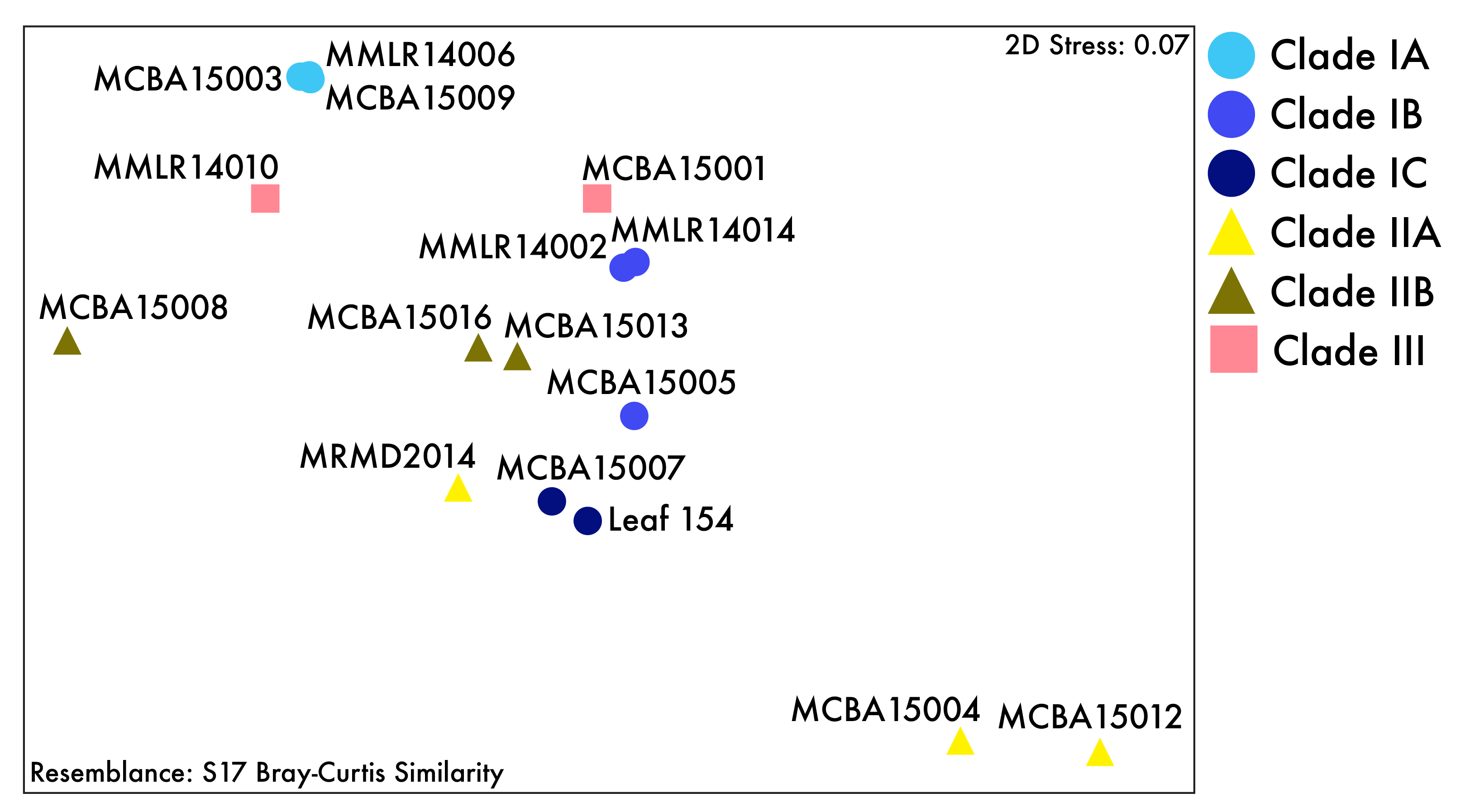

Supplement: FIG S4 [file mbo006173588sf4.tif]

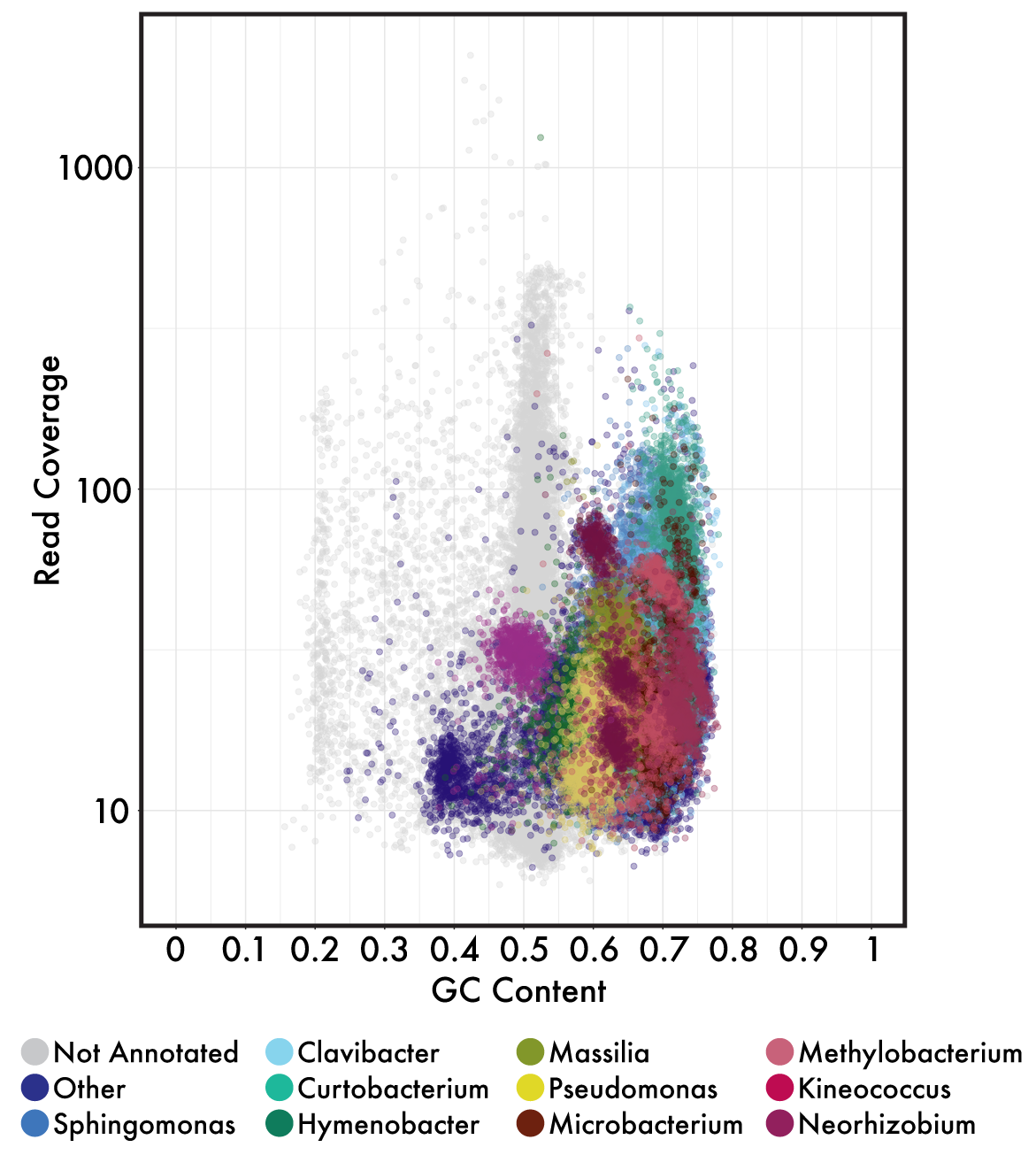

Supplement: FIG S5 [file mbo006173588sf5.tif]
