# Supplementary material for: Microdiversity of an Abundant Terrestrial Bacterium Encompasses Extensive Variation in Ecologically Relevant Traits
Source: mBio. 2017 Nov 14;8(6):e01809-17. doi: 10.1128/mBio.01809-17 (PMC5686540; doi:10.1128/mBio.01809-17)
Supplement: TABLE S1 [file mbo006173588st1.docx]

**Supplementary Table 1.** Average (± 1 SD) taxonomic relative abundance (%) of the bacterial communities across the metagenomic samples from 2010-2012 at the LRGCE.

| **Taxonomic Level** | **16S rRNA^a^** | **MG-RAST All Annotated Sequences** | **MG-RAST Annotated Marker Genes** | **Phylogenetic Classification using Marker Genes** |
| --- | --- | --- | --- | --- |
| **Phylum** |  |  |  |  |
| Actinobacteria | 51.29 ± 16.48 | 36.61 ± 7.24 | 55.41 ± 10.13 | 46.28 ± 7.71 |
| Bacteroidetes | 24.75 ± 10.24 | 8.32 ± 2.75 | 7.81 ± 3.60 | 9.13 ± 3.14 |
| Proteobacteria | 22.45 ± 9.68 | 44.47 ± 4.60 | 34.64 ± 7.14 | 33.44 ± 5.13 |
| **Family** |  |  |  |  |
| Microbacteriaceae | 37.21 ± 17.92 | 7.26 ± 2.73 | 25.11 ± 7.91 | 28.15 ± 7.23 |
| **Genus** |  |  |  |  |
| *Curtobacterium* | 18.64 | 0 | 0 | 7.77 ± 3.41 |
| **Clade** |  |  |  |  |
| Clade IA | - | - | - | 3.03 ± 1.36 |
| Clade IB | - | - | - | 0.58 ± 0.28 |
| Clade IC | - | - | - | 0.34 ± 0.17 |
| Clade IIA | - | - | - | 0.40 ± 0.21 |
| Clade IIB | - | - | - | 0.48 ± 0.21 |
| Clade III | - | - | - | 2.41 ± 1.26 |

^a^16S rRNA data referenced from Matulich *et al.* ISME. 2015.
