# Supplementary material for: Microdiversity of an Abundant Terrestrial Bacterium Encompasses Extensive Variation in Ecologically Relevant Traits
Source: mBio. 2017 Nov 14;8(6):e01809-17. doi: 10.1128/mBio.01809-17 (PMC5686540; doi:10.1128/mBio.01809-17)
Supplement: TABLE S2 [file mbo006173588st2.docx]

**Supplementary Table 2.** Genomic characterizations of all *Curtobacterium* isolates within individual clades.

|  | No. isolates | Genome size (Mbp) - range | ANI^a^ - range | AAI^b^ - range | AAI^b^ of 29 single-copy marker genes | No. total genes - range | No. core genes in clade | AAI^b^ of all clade-specific core genes - range |
| --- | --- | --- | --- | --- | --- | --- | --- | --- |
| Curtobacterium | 16 | 3.44 - 4.02 | 83.2 - 100 | 78.9 - 100 | 94.4 - 100 | 3191 - 3821 | 2128 | - |
| Clade IA | 3 | 3.60 - 3.65 | 99.2 - 100 | 99.4 - 100 | 99.9 - 100 | 3364 - 3430 | 3259 | 99.4 - 100 |
| Clade IB | 3 | 3.77 - 3.82 | 96.0 - 100 | 96.3 - 100 | 99.9 - 100 | 3534 - 3663 | 3124 | 96.6 -100 |
| Clade IC | 2 | 3.64 - 3.77 | 98.9 | 98.7 | 99.8 | 3441 - 3565 | 3249 | 98.9 |
| Clade IIA | 3 | 3.44 - 3.65 | 86.1 - 98.9 | 85.6 - 98.6 | 98.0 -100 | 3191 - 3433 | 2595 | 86.3 - 99.2 |
| Clade IIB | 3 | 3.95 - 4.02 | 86.8 - 98.4 | 87.4 - 98.6 | 98.0 -100 | 3703 - 3821 | 2997 | 88.1 - 98.9 |
| Clade III | 2 | 3.78 - 3.90 | 84.9 | 84.2 | 97.0 | 3599 - 3712 | 2796 | 84.7 |

^a^ANI = Average Nucleotide Identity; ^b^AAI = Average Amino Acid Identity
