# Supplementary material for: Microdiversity of an Abundant Terrestrial Bacterium Encompasses Extensive Variation in Ecologically Relevant Traits
Source: mBio. 2017 Nov 14;8(6):e01809-17. doi: 10.1128/mBio.01809-17 (PMC5686540; doi:10.1128/mBio.01809-17)
Supplement: TABLE S3 [file mbo006173588st3.docx]

**Supplementary Table 3.** Relative abundance (%) of *Curtobacterium* clades by treatment averaged (± 1 SD) across the metagenomic libraries from the LRGCE.

| **Taxonomic Level** | **Drought (R)** | **Nitrogen (N)** | **Control (C)** | **Percent Change R:C** | **Percent Change N:C** |
| --- | --- | --- | --- | --- | --- |
| **Genus** |  |  |  |  |  |
| *Curtobacterium* | 9.25 ± 3.98 | 6.37 ± 2.72 | 7.69 ± 2.99 | +20.19 | -17.16 |
| **Clade** |  |  |  |  |  |
| Clade IA | 3.49 ± 1.52 | 2.53 ± 1.07 | 3.06 ± 1.35 | +14.10 | -17.50 |
| Clade IB | 0.67 ± 0.32 | 0.46 ± 0.23 | 0.60 ± 0.27 | +11.58 | -23.85 |
| Clade IC | 0.39 ± 0.20 | 0.35 ± 0.15 | 0.29 ± 0.16 | +35.33 | +18.57 |
| Clade IIA | 0.47 ± 0.28 | 0.35 ± 0.15 | 0.37 ± 0.18 | +26.02 | -6.49 |
| Clade IIB | 0.55 ± 0.28 | 0.41 ± 0.16 | 0.48 ± 0.18 | +14.37 | -13.79 |
| Clade III | 3.00 ± 1.39 | 1.90 ± 1.00 | 2.36 ± 1.20 | +26.91 | -19.58 |
